# It's all about the sex, or is it? Humans, horses and temperament

**DOI:** 10.1371/journal.pone.0216699

**Published:** 2019-05-14

**Authors:** Kate Fenner, Georgina Caspar, Michelle Hyde, Cathrynne Henshall, Navneet Dhand, Fiona Probyn-Rapsey, Katherine Dashper, Andrew McLean, Paul McGreevy

**Affiliations:** 1 Sydney School of Veterinary Science, Faculty of Science, University of Sydney, Sydney, NSW, Australia; 2 Charles Sturt University, Wagga Wagga, NSW, Australia; 3 School of Humanities and Social Inquiry, Faculty of Law, Humanities and the Arts, University of Wollongong, Wollongong, NSW, Australia; 4 School of Events, Tourism and Hospitality Management, Leeds Beckett University, Leeds, United Kingdom; 5 Equitation Science International, Tuerong, Victoria, Australia; Institute of Animal Science, CZECH REPUBLIC

## Abstract

We propose that the anthropomorphic application of gender stereotypes to animals influences human-animal interactions and human expectations, often with negative consequences for female animals. An online survey was conducted to explore riders’ perceptions of horse temperament and suitability for ridden work, based on horse sex. The questionnaire asked respondents to allocate three hypothetical horses (a mare, gelding and stallion) to four riders compromising a woman, man, girl and boy. Riders were described as equally capable of riding each horse and each horse was described as suitable for all riders. Participants were also asked which horses (mares, geldings or stallions) were most suitable for the three equestrian disciplines of show-jumping, dressage and trail-riding. Logistic regression analyses were conducted to investigate people’s perceptions about suitability of horse types for particular riders, to evaluate if age, strength or gender were important in rider choice and to investigate riders’ allocation of various descriptors to a gelding, stallion or mare. There were 1,233 survey respondents, 94% of whom were female and 75% of whom were riders with at least eight years of experience. Binomial logistic regression revealed the girl had 2.5 times the odds of being allocated the gelding compared to the boy (*p* < 0.001). Respondents were significantly more likely to allocate the stallion to the man and nearly 50% of respondents did not allocate a horse to the boy, even though they ranked rider gender as least important to their choice (*p* < 0.001). In a forced choice selection of a positive or negative descriptor from a series of nine paired terms to describe horse temperament, a greater proportion of respondents assigned geldings positive ratings on terms such as calm, trainable, reliable and predictable. In terms of suitability for the three equestrian disciplines of show-jumping, dressage and trail-riding, participants overwhelmingly chose geldings for trail-riding, with mares being least preferred for both dressage and show-jumping disciplines. The results suggest that female riders are entering the horse-human dyad with gendered ideas about horse temperament and view horse-riding as an activity primarily for women and girls. This could have far-reaching implications for equine training and welfare.

## Introduction

Historically, horses have been used in war, agriculture, and transport [1] but more recently horse-riding has transitioned to a sporting and leisure activity with an associated shift in attitudes toward horses as companion animals [2, 3]. Today, opportunities to ride, own, handle and breed horses are readily available in many countries [4, 5]. With the horse’s transition from worker to companion, the proportion of women who spend time with horses has increased and human attitudes towards, and expectations of, the species have changed. Equine attributes that are now valued extend beyond the functionality of the horse and include specific temperament and personality traits [6, 7]. From the dressage arena to the Pony Club grounds, equids are purchased for their specific characteristics and temperament attributes [8].

Unlike companion dogs or cats that either remain as part of the same household their entire lives or are relinquished to shelters [9], horses are often seen as a commodity [10, 11]. The present day horse market is a liquid one that allows horses to be traded, sold, given away and even euthanized/killed with relative ease [12]. Excessive and unregulated breeding in many countries [13] has resulted in supply far exceeding demand [14], the consequences of which are often reflected in poor welfare outcomes for animals [15].

Today a horse buyer is faced with a number of choices pertaining to horses’ breed, age, sex, height, color and training experience. Seemingly the most straightforward of these choices is sex which is (anecdotally) often the first to be settled. Buyers can choose from a mare (intact female), a gelding (castrated male) or a stallion (entire male). Most leisure riders choose not to own stallions because of complicated housing and management issues, not least among which is the recurrent need to separate stallions from oestrous mares.

Scant published research exists on the effect of sex on equine trainability and personality attributes. Most studies report no differences in learning abilities or training outcomes between mares, geldings or stallions [16–22]. Temperament factors such as emotionality and fearfulness have been correlated with impaired learning in some studies [23, 24], but there are few reported data on how horse sex may affect the prevalence of such traits in domestic horses [25, 26]. Wolff et al. [27] found no effect of sex on emotionality in young horses in three handling tests and Kezierski et al. [28] reported that Arabian colts had higher heart rates than Arabian fillies during foundation training using a “conventional” method compared to a Natural Horsemanship method, where fillies’ heart rates were not significantly different from the colts. Sex differences in learning and behavior have been reported in young horses but learning tasks and therefore results vary. Yearling fillies appeared to learn at an accelerated rate during early training compared to male horses during two learning tests [29]. That said, a later study revealed that yearling fillies were reported by their student handlers as being more anxious, aggressive and reactive than geldings during a basic handling program but achieved similar training outcomes at the conclusion of the program [30]. When learning and training outcomes are assessed on the basis of the achievement of training milestones, sex differences are not reported (for example [26, 31–33]).

While convention dictates that younger riders should be mounted on more experienced horses, due to the presupposition that such horses are safer, due to having been exposed to more potentially aversive stimuli, and having more established responses to correct rider cues, there is an absence of scientific evidence to confirm if mares, gelding or stallions are better suited to riders of a given age or gender. In a preliminary study, Ille et al [34] found no differences in stress responses between horses ridden by male or female riders, suggesting perhaps that the gender of the rider may not matter to the horse. Previous studies that have explored a range of equestrian topics by surveying amateur riders have predominantly included women as respondents chiefly because there are more female riders at amateur level [35, 36]. However, in equestrian events at the professional level, there are more male riders [37] and in amateur and professional rodeo, more men than women participate in competitive rodeo activities [38]. The aim of the current study was to determine whether gender of a rider plays a role in ideas and beliefs about the temperaments and ridden behavior of mares, geldings and stallions.

## Materials and methods

Questionnaire: An online questionnaire was designed using the program SurveyMonkey (SurveyMonkey Inc., California, USA, www.surveymonkey.com) to gather information from horse owners and non-horse owners on four topics:

1. Preference for horse phenotypes. The results of this topic have been previously been published [39].
2. The suitability of horses for particular riders based on the sex of the horse and the gender and age of the rider.
3. Beliefs about perceived temperament characteristics of horses based on whether they are mares, geldings or stallions
4. Beliefs about the perceived suitability of mares, geldings and stallions for different equestrian pursuits. The results of this topic have previously been published [40]

The questionnaire presented participants with the following scenario:

“*You are left in charge of a well-known [Australian] Stock Horse stud which also runs a trail-riding centre*. *The stud is known for its reliable horses*. *The following four riders arrive for a trail ride without a booking*. *You assess them as all having the experience to ride any of the centre’s trail horses*. *There are only three horses available*, *so one person will miss out*.*”*

The horses that the participants could choose between were described as follows:

MARE, a 10-year-old Stock Horse mare
STALLION, a 10-year-old Stock Horse stallion
GELDING, a 10-year-old Stock Horse gelding

The riders that the participants could choose between were described as follows:

Man, Woman, Boy, Girl

Participants were asked to choose the most appropriate horse for each rider from the above list, using a forced ranking so one horse could be chosen for each rider and one person would fall under the ‘no horse’ category. Respondents were asked the following question:

Q: “Please choose the most appropriate horse for each rider (please note: This panel will allow you to select only three riders. Once the horse has been chosen, it cannot be allocated to another rider)”
Following this, they were asked to rate their decision in order of importance based on age, strength and gender of the rider (1 = Most important to 3 = Least important). Respondents were asked the following question:
Q: “When making your decision in Part A (matching riders with horses) please RANK the following in order of importance (1 is most important and 3 is least important- you can use each option ONCE)”

We were also interested in the terms that the participants associated with mares, geldings and stallions. Therefore, in a forced choice paradigm, participants were asked “In your opinion, which of these terms best describes most geldings?” This question was repeated for mares and stallions. These three questions were randomized, and terms presented as pairs in the following order: *Flighty* or *Calm*, *Unreliable* or *Reliable*, *Predictable* or *Unpredictable*, *Difficult* or *Easy*, *Trainable* or *Untrainable*, *Unwilling* or *Willing*, *Good attitude* or *Bad attitude*, *Bossy* or *Easy-going* and *Safe* or *Dangerous*.

To investigate whether there was a link between the sex of horses and the respondent’s association with different disciplines or recreational riding, participants were asked which horse, when given the choice of a gelding, stallion or mare, they would expect to be used for dressage and show-jumping and which horse would they choose for trail-riding.

Participants were asked to choose from one of the following statements to describe their involvement with horses: no experience with horse-riding, casual rider as a child only, casual rider as an adult, rider with at least 2 years’ experience, and rider with at least 8 years’ experience. Lastly, demographic information invited respondents to indicate their gender and age in years.

Participant enrolment: Advertisements were placed on website forums calling for participants in a “Horse Selection” survey. Forums included *Cyberhorse* (www.cyberhorse.com.au), *Horseyard* (www.horseyard.com.au) and *Bush Telegraph* (www.bushtelegraph.com). A web link was placed on the homepage of the [former] Faculty of Veterinary Science and the Human Animal Research Network at The University of Sydney. Two emails (an initial and a follow-up) with links to the survey were sent directly to Veterinary Science and Animal and Veterinary Bioscience undergraduate students at The University of Sydney’s Faculty of Veterinary Science requesting participation, regardless of whether students considered themselves experienced with horses. Approaches were also made to secretaries of the Australian Campdraft Association, Pony Club Association, Endurance Association, South Australian Dressage Association, Dressage NSW, National Pleasure Horse Association, Victorian Eventers Association and Horse Riding Clubs Association. In addition, twenty-seven national breed associations were also emailed to request the participation of members. The survey was also spread through social media channels (e.g. Facebook) and participants were asked to encourage others to take part and recruit a large variety of people, both with and without horse-riding and handling experience. While most websites were Australian based, the survey was not restricted to an Australian audience and respondents’ country of residence was not investigated. The survey opened on the 1st March 2012 and closed on the 1st June 2013.

This study was conducted under the approval of the University of Sydney Human Research Ethics Committee (approval number: 01-2010/12396).

### Statistical data analysis

Data were managed using Excel 2010 and then imported into SAS Statistical Program (Version 9.4 2002–2012 by SAS Institute Inc., Cary, NC, USA) for statistical analyses. Data were stacked to create one variable for ‘rider’ with four categories (boy, girl, man, woman) and one binary variable for each of the horse types (mare–yes/no; stallion–yes/no; and gelding–yes/no). Descriptive analyses were conducted by creating frequency tables and contingency tables of the variable ‘rider’ with each of the horse type variables. Binomial generalised linear mixed models were fitted using SAS Glimmix procedure to evaluate the association between ‘rider’ (fixed effect) and each horse type (outcome variables) to investigate people’s perceptions about suitability of horse types for particular riders. A de-identified participant code was included as a random effect to account for multiple observations per participant.

To investigate if age, strength or gender were important in choosing riders for horses, these variables were stacked to create two variables: ‘characteristic’ (with categories of strength, age and gender) and ‘importance’ (with categories of most, some and least). Multinomial generalised linear mixed models were fitted using SAS Glimmix procedure to investigate how the variable ‘characteristic’ (fixed effect) is associated with the variable ‘importance’ (outcome). Similar to above, the de-identified participant codes were included (as a random effect) to account for clustering.

The survey then investigated riders’ allocation of various descriptors to a gelding, stallion or mare. Descriptive analyses were conducted by creating frequency tables and contingency tables of the variable ‘horse sex’ with each of the descriptive variable pairs. Binomial generalised linear mixed model analyses were conducted to evaluate the association between the sex of the horse (fixed effect) and each descriptor (outcome variables) to investigate people’s perceptions about the personality traits of the different sexes of horse. A de-identified participant code was included as a random effect to account for multiple observations per participant.

The final section of the survey asked respondents to choose a gelding, stallion or mare for a variety of riding disciplines. Multinomial logistic regression analyses using the Logistic procedure were conducted to evaluate the effect of experience (explanatory variable) for nominating stallions, geldings and mares for trail ride, show-jumping and dressage (outcome variables).

## Results

### Participants

**Fig 1:**
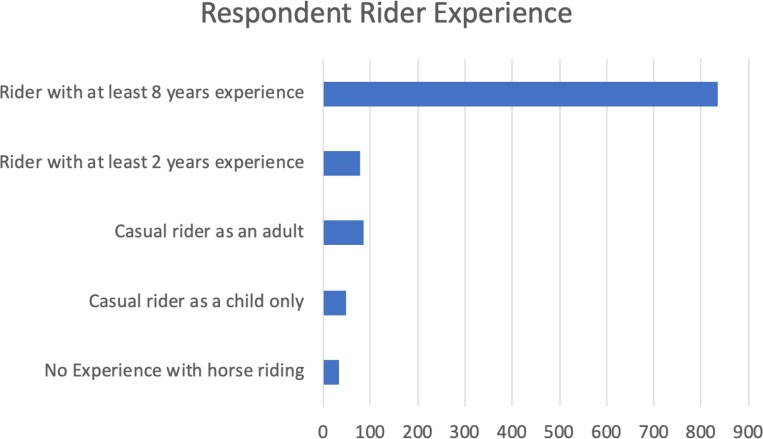
Respondent rider experience. Respondents’ (n = 1233) horse-riding experience. Riders with at least 8 years 77.46%, riders with at least 2 years’ experience 7.24%, casual rider as adult 7.88%, casual rider as child 4.45% and respondents with no experience with riding 2.97% (n = 1078).

One thousand two hundred and thirty-three (1233) people were surveyed. Of the respondents, 94% (n = 1159) were female. Descriptive data revealed the majority identified as ‘experienced’ horse riders (77% n = 949; see Fig 1 and Table 1).

**Table 1:** Respondents’ age in years and gender. Values in parentheses are row percentages.

Age (years)	Female	Male	Total
18–30	380 (96%)	15 (4%)	395
31–45	301 (94%)	20 (6%)	321
46–60	216 (91%)	22 (9%)	238
61–80	39 (89%)	5 (11%)	44

Females represented 94% of respondents and 96% of all respondents were aged between 18 and 60 years.

### Horse allocation

Respondents were asked to assign a gelding, stallion or mare to the man, woman, boy or girl, leaving one rider with no horse assigned.

More than half of the respondents allocated the gelding to the girl. The girl had 2.5 times the odds of being allocated the gelding than the boy (Table 2).

**Table 2:** Horse allocation odds ratio estimates for geldings, stallions and mares.

Variable	Gender	Odds ratio	95% Cl	*p-value*
**Gelding**				<0.001
	Boy*	1.00		
	Girl	2.53	2.14, 2.98	
	Man	0.21	0.17, 0.27	
	Woman	0.30	0.24, 0.37	
**Stallion**				<0.001
	Boy*	1.00		
	Girl	2.50	1.3, 4.8	
	Man	104.00	59.5, 181.6	
	Woman	72.40	41.4, 126.5	
**Mare**				<0.001
	Boy*	1.00		
	Girl	1.99	1.66, 2.39	
	Man	0.59	0.47, 0.73	
	Woman	1.99	1.66, 2.39	

*Reference category

Respondents (n = 1233) assigned the gelding to the boy 29% of the time and the girl was 2.5 times more likely to be allocated the gelding rather than the boy. Almost all respondents assigned the stallion to one of the adults, with the man having 104 times the odds of being allocated the stallion over the boy and the woman 72 times the odds of being allocated the stallion over the boy. When asked to allocate the mare to rider, both the girl and the woman had twice the odds of being allocated the mare over the boy or the man.

**Fig 2:**
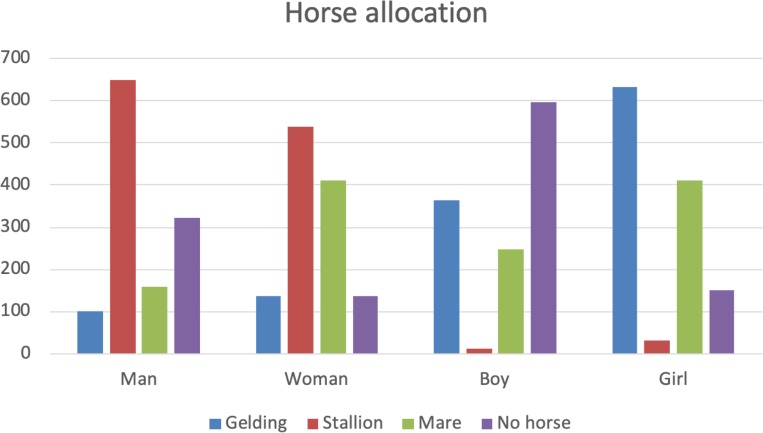
Horse allocation. Respondents (n = 1233) assigned either a gelding, stallion or mare to the man, woman, boy and girl, leaving one rider without a horse. The man was not allocated a horse twice as often as the woman and the girl and the boy was not allocated a horse most frequently.

The decision was the clearest when it came to deployment (or otherwise) of the stallion, with the adults being allocated that horse by almost all respondents and the man being given the stallion more often than the woman (see Fig 2). Neither of the children was allocated the stallion to ride, other than by a handful of respondents (see Fig 2).

For selection of a rider for the stallion, the man had 104 times the odds of being selected over the boy and the woman 72 times the odds of being selected over the boy (Table 2).

Human gender had a significant influence on responses when participants allocated the mare. Both the girl and the woman had twice the odds of being allocated the mare over the boy or the man (Table 2).

**Fig 3:**
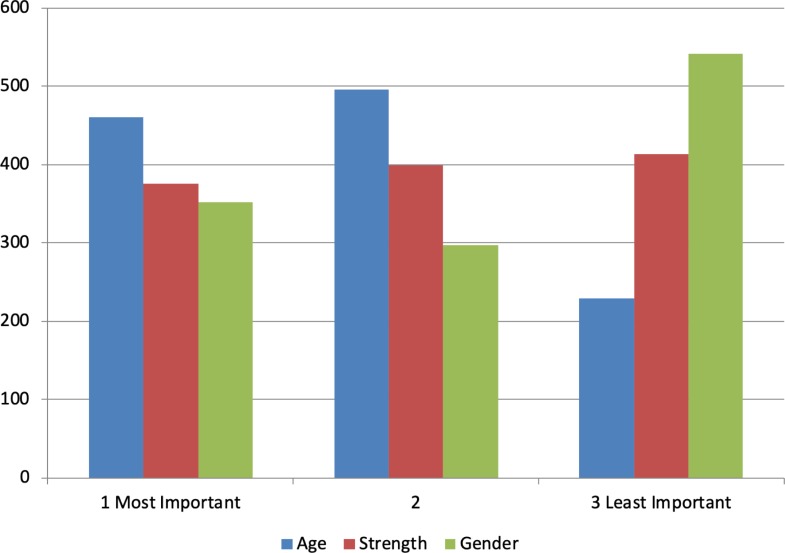
Allocation considerations. Fig 3: When allocating horses to riders, respondents were asked how important the riders’ age, strength and gender were in their decision-making process. 40% of respondents nominated age as the most important consideration.

Approximately 40% of the respondents nominated age as their most important consideration when allocating riders to horses, whereas about 30% each nominated strength and gender as the most important decision-making characteristics for allocating horses for riders (see Fig 3). Logistic regression analyses indicated that respondents were about twice as likely to give importance to age over strength, with age having 2.24 times the odds ratio of gender, and 1.37 times the odds ratio of strength, when respondents considered horse allocation.

### Horse temperament descriptors

**Fig 4:**
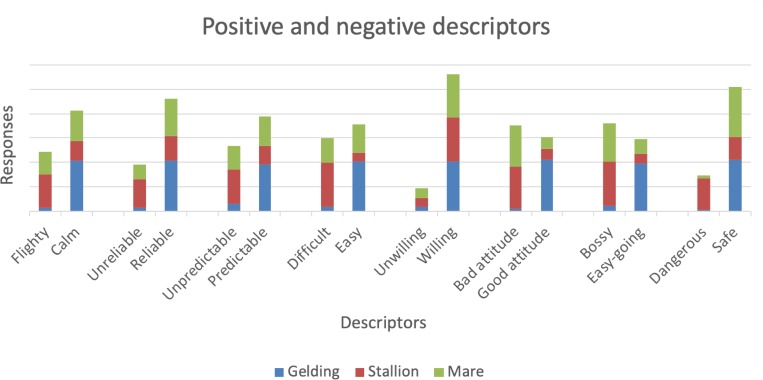
Positive and negative descriptors assigned to geldings, stallions and mares. More than 90% of respondents (n = 1090) allocated geldings positive descriptors. Stallions received the least positive attributes.

Respondents were required to assign one adjective of a dichotomour pair as an indicative attribute of gelding, stallion and mare. The results are presented in Fig 4. More than 90% of respondents classified geldings as *Calm*, *Reliable*, *Easy*, *Trainable*, *Willing*, having a *Good attitude*, *Easy*-*going* and *Safe*, with over 86% also saying they were *Predictable* (Table 3). The respondents considered stallions to be *Trainable* with *Good attitudes* but, at the same time, *Bossy* and *Difficult*. Mares scored highly as *Safe* and *Trainable*, but respondents were less sure about assigning them attributes such as *Easy-going*, *Predictable* or *Reliable*. Also, mares were considered to be *Bossy* with 80% of respondents assigning this attribute to them.

**Table 3:** Odds ratio estimates for horse descriptor allocation.

Description	Sex				odds ratio	95% CL		Probability		P-value
**Flighty/calm**		**Flighty**	Calm*	**Total**						**<0.001**
	Gelding	67	1023	1090	26	19.9, 34.0		0.94		
	Mare	464	626	1090	2.3	1.9, 2.7		0.57		
	Stallion	691	406	1097	1			0.37		
**Unreliable/**		**Unreliable**	Reliable*	**Total**						
**reliable**	Gelding	54	1036	1090	23	17.2, 30.6		0.95		
	Mare	310	780	1090	3	2.6, 3.6		0.72		
	Stallion	598	499	1097	1			0.46		
**Difficult/easy**		**Difficult**	Easy*	**Total**						**<0.001**
	Gelding	85	1005	1090	54	41.5, 70.4		0.92		
	Mare	517	573	1090	5.1	4.2, 6.1		0.53		
	Stallion	900	197	1097	1			0.18		
**Unwilling/**		**Unwilling**	Willing*	**Total**						**<0.001**
**willing**	Gelding	87	1003	1090	2.3	1.8, 2.9		0.92		
	Mare	192	898	1090	0.9	0.8, 1.1		0.82		
	Stallion	181	916	1097	1			0.84		
**Bossy/Easy-going**		**Bossy**	Easy-going*	**Total**						**<0.001**
	Gelding	112	978	1090	38.5	30.1, 49.2		0.89		
	Mare	802	288	1090	1.6	1.3, 1.9		0.26		
	Stallion	894	203	1097	1			0.19		
**Predictable/**		Predictable*	**Unpredictable**	**Total**						**<0.001**
**unpredictable**	Gelding	147	942	1089	11.6	9.5, 2.0		0.87		
	Mare	474	616	1090	2.4	2.0, 2.8		0.57		
	Stallion	390	707	1097	1			0.36		
**Trainable/**		Trainable*	**Untrainable**	**Total**						**0.604**
**untrainable**	Gelding	1042	48	1090	1.1	0.7, 1.5		0.96		
	Mare	1033	57	1090	0.9	0.6, 1.3		0.95		
	Stallion	1046	51	1097	1			0.95		
**Good/bad attitude**		Good*	**Bad**	**Total**						**<0.001**
	Gelding	1046	44	1090	6.6	4.8, 9.0		0.96		
	Mare	851	239	1090	0.9	0.8, 1.2		0.78		
	Stallion	859	238	1097	1			0.78		
**Safe/dangerous**		Safe*	**Dangerous**	**Total**						**<0.001**
	Gelding	1058	32	1090	44.2	31.2, 62.7		0.97		
	Mare	1012	78	1090	17.4	13.7, 22.0		0.93		
	Stallion	469	628	1097	1			0.43		

Respondents (n = 1090) were asked to assign either a positive or negative temperament descriptor to a gelding, stallion and mare. The geldings received the most positive descriptors.

Missing data: This survey item was not completed for geldings and mares by some respondents, as indicated in the total number of responses column.

CL: Confidence limits; SE: Standard error.

*All probabilities are calculated for the positive temperament trait for all variables

### Horse choice by discipline

**Fig 5:**
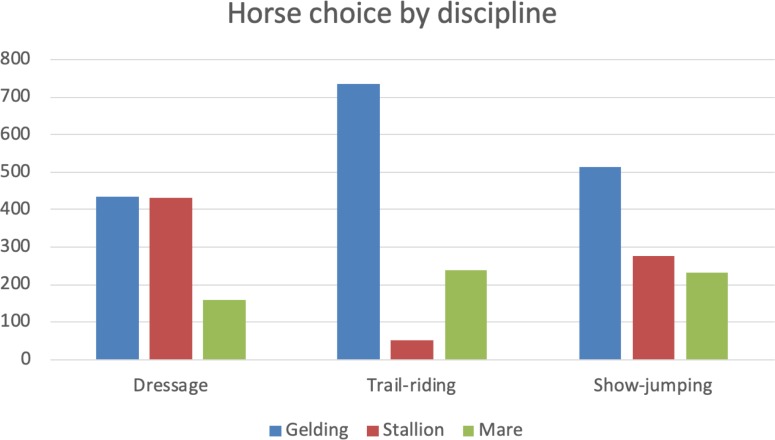
Horse choice by discipline. Respondents (n = 1230) were asked whether they were more likely to see a gelding, stallion or mare competing in Dressage and show-jumping and which sex of horse they would choose for trail-riding. Geldings were preferred over mares across all disciplines.

Respondents were then asked which horses would be most likely to be seen competing in Dressage and show-jumping and, when given the choice of a gelding, stallion or mare, which horse the respondent would chose for trail-riding (see Fig 5).

Stallions and geldings were nominated as equally suitable for dressage by 42.1–42.6% of respondents respectively, with 15.3% selecting mares. Most of the respondents, 71.8%, nominated a gelding for trail-riding, whereas 23% chose mares and just 5% chose stallions. For show-jumping, 50% of respondents nominated a gelding, with the remainder being roughly divided between stallion (27.2%) and mares (22.2%). Compared to stallions, geldings were about eight times (odds ratio: 7.75; 95% CI: 5.68, 10.77) and mares were about six times (odds ratio: 5.6; 95% CI: 3.96, 7.96) more likely to be nominated for trail ride than for show-jumping. On the other hand, both geldings and mares were less likely than stallions to be nominated for dressage than for show jumping (odds ratio gelding vs. stallion: 0.55, 95% CI: 0.45, 0.66; mare vs. stallion: 0.44, 95% CI: 0.34, 0.56).

**Fig 6:**
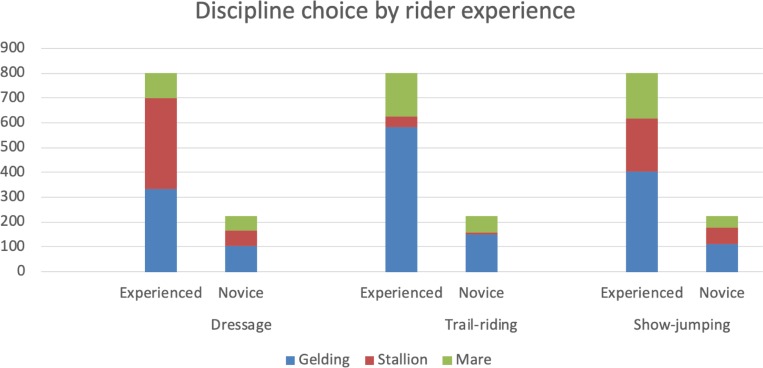
Respondents (n = 1230) were asked which horse they would expect to see in Dressage and show-jumping and which horse they would choose to ride on a trail ride. The figure shows discipline choice by rider experience level.

Respondents with more riding experience were more likely to expect to see a stallion in the dressage arena and riders of all experience levels chose a gelding for trail-riding purposes (see Fig 6).

Experienced riders were significantly more likely to expect to see a stallion competing in the dressage arena compared to a gelding (odds ratio: 1.77, 95% CI: 1.45, 2.16) or a mare (odds ratio: 3.14, 95% CI: 2.46, 4.00). For trail-ride, experienced riders were more likely to expect to see a stallion (odds ratio: 1.68, 95% CI: 1.09, 2.68) or a gelding (odds ratio: 1.39, 95% CI: 1.14, 1.69) compared to a mare. However, experience did not seem to influence preferences for show jumping (p = 0.30).

## Discussion

Our results suggest that participants in this study, who were mainly female (see Table 1), hold preconceived ideas about horse temperament and suitability based on the sex of the horse and the age and gender of the rider. The large proportion of female respondents in this study accurately reflects the gender distribution of riders in Australia, as found in many other studies [41–44].

Horse-rider allocation decisions must have been made based on rider gender, age and horse sex because the questionnaire described each horse as being suitable for any of the riders. It is worth noting that several respondents objected to being forced to decide based on the limited information provided. Under these circumstances, one might expect that the age and gender of the person who misses out on riding should be randomly distributed, in that there should be an equal probability of boy/girl or man/woman not being allocated a horse and equal probability of each horse being assigned to each rider. Clearly, our results were significantly skewed as a function of respondents’ bias. Predictably, the stallion was almost always allocated to an adult, and preferentially, the man. The gelding was most often allocated to a child, with the girl being assigned the gelding more often than the boy and the mare more likely to be assigned to the woman or the girl. The most unexpected finding in this section of the survey was that the boy was not allocated a horse to ride by almost half of the respondents. When asked to explain their choices, these same respondents ranked the hypothetical riders’ gender as the least important factor in their decision-making process, with age being ranked as most important, followed by strength.

There is a clear disconnect between respondents’ actual choices and the factors they cite as important when matching horses and riders. Preference for female riders appears to extend to the adults, with the man failing to be allocated a ride twice as often as either the girl or the woman. These data appear to reflect the predominance of women in recreational horse activities [42] and, given that 94% were female and 77% had eight or more years of riding experience, may reflect personal preferences based on the respondents’ own experiences of horse-riding. Horse-riding activities were reported to increase self-esteem and general self-efficacy (a putative measure of one’s beliefs about one’s performance capabilities in particular situations) in women and girls in a survey of Norwegian riders [45]. We might speculate that the choice of the boy or man to miss out on the hypothetical ride could reflect the respondents’ assumptions about the likely level of interest or motivation to ride held by the males, based on the respondents’ own experiences. Among Australian children, girls participate in equestrian sports at substantially higher rates than boys [43]. The selection of the female rider instead of the man may reflect the dominance of women in horse-riding, its identification with women and the ways in which women privilege the transfer of horse-riding skills from one generation of women to the next. In short, it is likely that the (mainly female) respondents see the girl as the ‘rider’ and as the more enthusiastic apprentice to equestrian sports, thereby perpetuating the predominance of women in the sport overall. Women’s predominance in equestrian sports may well also relate to broader sociological observations about women’s attitudes to horses and animals more generally [44]. It may also result from anecdotal beliefs that females are better equipped to handle horses and particularly female horses, on account of gender attributes such as empathy, risk-aversion, altruism and patience which have been identified in female gender stereotypes in multiple countries across varying economic situations and activities [46–48]. Conversely, this result may reflect beliefs that young males have less impulse control and are more inclined to engage in sensation-seeking behavior [49] which could place both the boy and the horse at risk of harm. While the data do not tell us which of these factors (if any) play a role in the decision, it is clear that there is a consistency of belief among the current respondents about the girl having the opportunity to ride the horse before the boy.

Further stereotypes and bias were encountered in the current study when respondents were invited to choose between dichotomous adjectives to characterize mares, geldings and stallions. The results for geldings were clear and they were positively classified in each of the nine categories by almost all respondents. Positive and negative attributes were mostly evenly spread for mares, with *Bossy* and *Bad* being the only negative factors significantly attributed to them. Stallions scored very highly on *Trainability*, but at the same time were considered *Difficult*, *Bossy* and *Dangerous*. These results suggest that female participants enter the horse-human dyad with specific ideas based on the sex of the horse. Similar findings were reported when these same participants provided short text answers concerning their horse choice for particular disciplines [40]. We could also speculate that this set of ideas is also being transmitted from woman to girl riders and is part and parcel of the culture of horse-riding that sees horse-riding as a sport for girls and women, rather than for men and boys.

But just how accurate is this set of ideas that is being transmitted? Given that most studies of equine learning and temperament do not report sex influences on horse temperament, trainability or learning ability, including between geldings and stallions or mares and stallions, the reason respondents assigned the term *Bossy* to mares and stallions but not geldings appears to reside in beliefs and is yet to be explored experimentally. This bias may reflect the respondents’ gendered interpretations of past encounters with male and female horses, in which horse behavior was identified as resulting from the influence (or lack of influence, in the case of geldings) of sex hormones, rather than other causes such as pain [50], training confusion [51] or rider failures [52]. While little research has yet been undertaken investigating the role that sex hormones play in riding and competing with stallions and mares, there is anecdotal evidence that stallions can become difficult to control, notably in the presence of mares in oestrus. Owner gender and animal sex are reported to influence the interpretations of companion cat and dog behavior, including the behavior of de-sexed animals [53, 54]. Indeed, in male dogs this is an area of scientific enquiry that continues to yield surprising results with desexing appearing to exacerbate many behaviors that were thought to be ameliorated by it [55].

Assuming the horse is behaving in a particular way based on its sex alone may lead riders, trainers and handlers to erroneous conclusions about horse behavior and a consequent failure to address the etiology of unwanted behavior. Riders are in a position to exert a significant influence over factors that affect horse behavior such as their individual riding skills, equipment use and the physical health of the horse [50, 52, 56]. Sex-based assumptions exclude other possible causes of any unwanted behaviors, thereby limiting the riders’ ability to be proactive in their interactions with their mounts. If the behavior of mares and stallions is interpreted as arising from gendered beliefs, rather than other causes, they may be at risk of having stress or pain-related behaviors ignored because of this bias.

The attribute *Bossy*, which the current participants used to characterize both mares and stallions, is of concern. The concepts of leadership and dominance are still commonly applied in horse training contexts and may encourage or justify the application of punishment [57–59]. Especially prevalent in Natural Horsemanship (NH) training philosophies, the dominance hierarchy view of human-horse interactions places the trainer as a herd leader with the horse required to be a submissive participant [60]. In addition, many NH practitioners state that feral horse herds are organized around a dominant “alpha” mare who directs and controls the activities of the herd, including the stallion [59–61]. Under such conditions the *Bossy* horse is at risk of having any undesirable behavior interpreted as a lack of respect or as a hierarchical challenge rather than fear, pain or confusion. Such an interpretation can lead directly to positive punishment of the unwanted behavior rather than diagnosis of its cause. It is possible that sex hormones may influence a horse’s tendency to trial or not trial a correct response during training and this could be interpreted as *Bossy* behavior. The combination of bias and stereotyping will shape relationships with horses and likely have a detrimental effect on welfare if underlying pathologies or training failures are not addressed [50, 62].

A limitation of the current study is that respondents were required to choose between attributes which were selected by the authors. As such, respondents could not indicate if they did not believe that either attribute in each pair accurately reflected an equine sex-based attribute. Additionally, respondents could not choose more than one category of horse for use in each discipline, so the results may not accurately reflect their views about the relative, rather than absolute, suitability of mares, geldings and stallions for each equestrian activity. The questionnaire gave no details on whether the hypothetical mare was in oestrus, a reproductive state that may sequentially increase and then decrease a mare’s inclination to approach other horses and influence the hypothetical stallion’s interest in the mare [63]. The frequent nomination of the gelding for trail-riding may reflect an expectation of reliable and predictable horse behavior arising from the relative absence of sex hormones. Additionally, if undertaken in the company of other horses, the perceived reduction of sex-hormone influences over intraspecific behavior during trail-riding could contribute to perceptions of safety for riders.

These same respondents were asked to give short answers to questions surrounding their choice of a mare, gelding or stallion for the disciplines of dressage, show-jumping and trail-riding. The results of these qualitative data were the subject of further study [40]. Dashper et al (2018) also reported an overall preference for male horses, with mares selected less than twenty-five percent of the time when asked to choose a horse for a sport or leisure activity. The reasons given by riders for not selecting mares centered around the belief that mares were not consistent in their behavior and the choice of a mare was often tempered with the statement that one needed a ‘good mare’ or ‘needed to happen across her on a good day’[40].

The attribution of gendered characteristics onto horse behavior by female respondents suggests that they may default to attributing undesirable horse behavior to gender, rather than factors such as pain or training confusion. This attribution may hinder riders’ seeking appropriate remedies for unwanted behavior in their mares or stallions. Further research into the attitudes of male riders towards mares, geldings and stallions could confirm if such views are shared by male riders too. Work in other species has identified gender and sex-based interpretations of behavior by both male and female owners of companion animals such as dogs and cats [54] and further observational research also could explore whether the gendered understandings are replicated when owners handle and ride horses. Furthermore, there appears to be a disconnect between owners’ attitudes to their horses based on the sex of the horse and the findings of learning, training and temperament studies which, to date, have not identified significant sex-based differences in learning abilities, temperament traits or training outcomes in mature horses and find contradictory effects of sex on training outcomes in young horses reviewed [64]. Additionally, research to investigate differences in equine learning, behavior or performance outcomes when ridden by males and females merit empirical study. In preferring male horses, and particularly geldings for most equestrian activities, riders may be unnecessarily limiting their options by avoiding mares which current evidences suggests are no less likely to achieve training outcomes and no more likely to possess emotional or fearful temperaments than geldings.

## Conclusions

Gender, behavior and sex stereotyping are prevalent in the equestrian industries. Female riders appear to be entering the horse-human dyad with preconceived gendered ideas about horse temperament and view horse riding as a sport for females. The current survey of human preferences for certain horses prompted more responses from women than from men. This reflects the predominance of women in most equestrian activities. Women riders express a preference for combining female riders with castrated male horses. Castrated male horses were also preferred for each equestrian discipline of show-jumping, dressage and trail-riding. Mares are perceived, largely without scientific foundation, as being less reliable, less predictable and less desirable than their castrated male counterparts. In some cases, this is likely to compromise mare welfare.
